# SepA Enhances *Shigella* Invasion of Epithelial Cells by Degrading Alpha-1 Antitrypsin and Producing a Neutrophil Chemoattractant

**DOI:** 10.1128/mBio.02833-21

**Published:** 2021-11-02

**Authors:** Mario Meza-Segura, James R. Birtley, Ana Maldonado-Contreras, Christian Mueller, Karl J. Simin, Lawrence J. Stern, Beth A. McCormick

**Affiliations:** a Department of Microbiology and Physiological Systems, University of Massachusetts Medical School, Worcester, Massachusetts, USA; b Program in Microbiome Dynamics, University of Massachusetts Medical School, Worcester, Massachusetts, USA; c Department of Pathology, University of Massachusetts Medical School, Worcester, Massachusetts, USA; d Horae Gene Therapy Center, University of Massachusetts Medical School, Worcester, Massachusetts, USA; e Department of Molecular, Cell and Cancer Biology, University of Massachusetts Medical School, Worcester, Massachusetts, USA; School of Medicine, Oregon Health and Science University

**Keywords:** *Shigella*, alpha-1 antitrypsin, chemotaxis, gut inflammation, neutrophils

## Abstract

*Shigella* spp. are highly adapted pathogens that cause bacillary dysentery in human and nonhuman primates. An unusual feature of *Shigella* pathogenesis is that this organism invades the colonic epithelia from the basolateral pole. Therefore, it has evolved the ability to disrupt the intestinal epithelial barrier to reach the basolateral surface. We have shown previously that the secreted serine protease A (SepA), which belongs to the family of serine protease autotransporters of *Enterobacteriaceae*, is responsible for the initial destabilization of the intestinal epithelial barrier that facilitates *Shigella* invasion. However, the mechanisms used by SepA to regulate this process remain unknown. To investigate the protein targets cleaved by SepA in the intestinal epithelium, we incubated a sample of homogenized human colon with purified SepA or with a catalytically inactive mutant of this protease. We discovered that SepA targets an array of 18 different proteins, including alpha-1 antitrypsin (AAT), a major circulating serine proteinase inhibitor in humans. In contrast to other serine proteases, SepA cleaved AAT without forming an inhibiting complex, which resulted in the generation of a neutrophil chemoattractant. We demonstrated that the products of the AAT-SepA reaction induce a mild but significant increase in neutrophil transepithelial migration *in vitro*. Moreover, the presence of AAT during *Shigella* infection stimulated neutrophil migration and dramatically enhanced the number of bacteria invading the intestinal epithelium in a SepA-dependent manner. We conclude that by cleaving AAT, SepA releases a chemoattractant that promotes neutrophil migration, which in turn disrupts the intestinal epithelial barrier to enable *Shigella* invasion.

## INTRODUCTION

Species from the *Shigella* genus are Gram-negative, highly infective, and primate-restricted pathogens that infect the intestinal colonic epithelium (1). *Shigella* infection, known as shigellosis, results in an acute inflammatory disease of the intestine associated with fever, nausea, anorexia, dehydration, tenesmus, and mucopurulent bloody diarrhea (1). Globally, *Shigella* is responsible for between 80 and 165 million cases of diarrhea, accounting for nearly 600,000 deaths per year (2, 3), constituting the second leading cause of death due to diarrheal disease (4).

Four different *Shigella* species have been defined based on their biochemical, serological, and clinical phenotypes: S. dysenteriae (serogroup A), S. flexneri (serogroup B), S. boydii (serogroup C), and S. sonnei (serogroup D) (5). Among them, S. dysenteriae is responsible for epidemic disease outbreaks and the most severe form of dysentery, which causes the majority of fatal shigellosis cases (6). On the other hand, S. flexneri is the most prevalent worldwide and accounts for most cases of endemic shigellosis in developing countries, affecting mainly children under 5 years of age (1). *Shigella* strains are characterized by possessing a large virulence plasmid containing a 31-kb region (Mxi-Spa locus) shown to be necessary and sufficient for the invasion of epithelial cells and macrophage killing (6). This region harbors genes encoding proteins essential for the assembly and function of a type III secretion system (T3SS), including translocators (IpaB, IpaC, and IpaD), effectors (IpaA, IcsB, IpgB1, and IpgD), and transcriptional activators (VirB and MxiE) (7).

*Shigella* has a distinctive mode of pathogenesis that involves the invasion of colonic epithelial cells through their basolateral pole or submucosa, instead of the apical pole facing the intestinal lumen (8). Therefore, *Shigella* has evolved the ability to become translocated from the intestinal lumen to the submucosa. Two primary mechanisms have been put forward to explain how *Shigella* acquires its basolateral position. The first proposes that *Shigella* undergoes transcytosis of the epithelium via M cells (9). The second mechanism posits that *Shigella* creates a breach in the colonic epithelium to facilitate paracellular transit into the submucosa (10–12). *Shigella* generates this breach directly by disrupting the intercellular junctions (10) or destabilizing the intestinal epithelial barrier (12) or indirectly by stimulating the transepithelial migration of neutrophils from the submucosa to the intestinal lumen, which ultimately opens up intercellular spaces that allow *Shigella* paracellular transit (11).

To more deeply understand the mechanisms underlying *Shigella* pathogenesis, we studied the role of a protease encoded on the virulence plasmid, the secreted serine protease A (SepA), and found that this protein is crucial for the initial destabilization of the epithelial integrity (12). SepA is a member of the superfamily of serine protease autotransporters of *Enterobacteriaceae* (SPATE), a group of virulence factors widely expressed by enteric pathogens, including *Shigella*, Escherichia coli, and Salmonella (13). In addition to secreting SepA, *Shigella* secretes two more SPATES encoded on its chromosome: the protein involved in colonization (Pic) and the *Shigella* IgA-like protease homolog (SigA) (13). Uniquely, SepA is the major protein secreted by S. flexneri in culture (14) and is also highly secreted during infection (15, 16). We determined that during apical S. flexneri infection of colonic epithelium, SepA activates cofilin, a major actin polymerization factor (12). Cofilin is an actin-binding protein that controls the depolymerization of actin filaments and promotes the opening of tight junctions in the intestinal epithelium, resulting in increased barrier permeability (17, 18). We further showed that SepA-dependent alteration of the epithelial barrier is necessary for S. flexneri invasion of epithelial cells and that it is coupled to a strong induction of neutrophil transepithelial migration (12). In this regard, the alteration of the intestinal barrier integrity has also been associated by other groups with the enterotoxin activity of SepA (14, 19).

Despite the fundamental implication of SepA on *Shigella* invasion (12, 14), particularly in the activation of cofilin (12), the direct targets of this protease are still unknown. In this study, we identify alpha-1 antitrypsin (AAT), the archetype member of a family of serine proteinase inhibitors (SERPIN) (20), as the first known target of SepA. Additionally, we describe a mechanism whereby the cleavage products of AAT directly drive the transepithelial migration of neutrophils, a process that also facilitates *Shigella* infection (11).

## RESULTS

### SepA targets a myriad of proteins involved in intestinal epithelial barrier integrity.

We have shown previously that SepA facilitates the bacterial transit of S. flexneri from the apical to the basolateral surface of the epithelium by disrupting the intestinal epithelial barrier (12). To investigate the protein targets cleaved by SepA in the intestinal epithelium, we incubated a sample of homogenized human colon with purified wild-type (WT) SepA or with a catalytically inactive mutant of this protease, S211A SepA. Cleaved proteins were identified using two-dimensional difference in gel electrophoresis (2D-DIGE) with in-gel trypsin digestion of differentially labeled spots and tandem mass spectrometry (MS/MS) characterization of products.

Cy3 and Cy5 were used to label WT SepA-treated and S211A mutant-treated homogenized human colon preparations, respectively (see Fig. S1 in the supplemental material). The image with overlaid Cy3 and Cy5 dyes showed 24 unique spots only in WT SepA-treated homogenized human colon preparations. MS/MS analysis of these unique spots corresponded to 18 different proteins (Table 1), revealing that SepA targets an array of proteins involved in diverse cellular processes, including cytoskeletal organization (gamma-actin, alpha-actinin, and IQGAP1), cell-to-cell interactions (cofilin-1 and galectin-4), mucin production (anterior gradient protein 2 homolog), iron homeostasis (ferritin light chain), regulation of transcription (heterogeneous nuclear ribonucleoprotein A2/B1 and histidine triad nucleotide-binding protein 1), RNA trafficking (heterogeneous nuclear ribonucleoprotein A3), synthesis of glycans (*N*-acetyl-d-glucosamine kinase), degradation of reactive oxygen species (ROS) (peroxiredoxin-2), protein folding (calreticulin), and the inhibition of serine proteases (AAT). This observation is consistent with those of previous studies showing that other S. flexneri SPATE, including Pic and SigA, can degrade multiple different substrates (13, 21).

**TABLE 1:** SepA targets identified by tandem mass spectrometry

Spot	Protein name (entry name, UniProt)	Function (reference)
12	2,4-Dienoyl-coenzyme A reductase, mitochondrial (DECR_HUMAN)	Mitochondrial enzyme required for the β-oxidation of unsaturated fatty acids (86)
23	Gamma-actin (ACTG_HUMAN)	Globular multifunctional protein that constitutes the monomeric unit of cytoskeletal microfilaments (87)
1, 2	Alpha actinin-4 (ACTN4_HUMAN)	Cytoskeletal protein that participates in the organization of cell cytoskeleton and is adjacent to adherent junctions (88)
5, 6, 24	Alpha-1 antitrypsin (A1AT_HUMAN)	Serin protease inhibitor important for the inhibition of neutrophil elastase at inflammatory sites to abate incidental destruction of surrounding tissue and to facilitate tissue repair (49)
8, 9	Annexin A2 (ANXA2_HUMAN)	Multifunctional Ca^2+^ and phospholipid-binding protein involved in the activation of plasmin, the regulation of membrane dynamic events, and the regulation of inflammatory processes (89)
21	Anterior gradient protein 2 homolog (AGR2_HUMAN)	Protein disulfide isomerase involved in protein quality control in the endoplasmic reticulum and essential for the production of intestinal mucus (36, 90)
10	Calreticulin (CALR_HUMAN)	Multifunctional Ca^2+^-binding protein that operates as a chaperone to assist correct protein folding in the endoplasmic reticulum (91)
20	Cofilin-1 (COF1_HUMAN)	Actin-binding protein that controls the depolymerization of actin filaments and promotes the opening of tight junctions in the intestinal epithelium (17)
18	Ferritin light chain (FRIL_HUMAN)	Light subunit of the ferritin protein, important for iron homeostasis (92)
11, 19	Galectin-4 (LEG4_HUMAN)	Beta-galactoside-binding protein implicated in cell proliferation, apoptosis, differentiation, and intercellular adhesion (93)
17	Heterogeneous nuclear ribonucleoprotein A2/B1 (ROA2_HUMAN)	RNA-binding protein involved in the transcription, splicing, transport, stability, and translation of a large no. of genes (94)
13	Heterogeneous nuclear ribonucleoprotein A3 (ROA3_HUMAN)	RNA-binding protein that binds the *cis*-acting response element A2RE and participates in the cytoplasmic trafficking of RNA (95)
22	Histidine triad nucleotide-binding protein 1 (HINT1_HUMAN)	Phosphoramidase that regulates the activities of different transcription factors and participates in the regulation of apoptotic pathways (96, 97)
3, 4	Ras GTPase-activating-like protein IQGAP1 (IQGA1_HUMAN)	Scaffold protein that participates in the modulation of the cytoskeletal architecture, cytokinesis, intracellular signaling, and intercellular interactions (98)
7	*N-*Acetyl-d-glucosamine kinase (NAGK_HUMAN)	Sugar kinase necessary to catalyze the conversion of *N*-acetylglucosamine (GlcNAc) to GlcNAc-6-phosphate, a component that eventually leads to the synthesis of O-/N-glycans and sialic acid (99)
16	Peroxiredoxin-2 (PRDX2_HUMAN)	Antioxidant enzyme that hydrolyzes hydrogen peroxide molecules to water and protects cells against oxidative damage from reactive oxygen species (ROS) (100)
15	Protein/nucleic acid deglycase DJ-1 (PARK7_HUMAN)	Redox-sensitive molecular chaperone that inhibits protein aggregation and helps to regulate oxidative stress (101, 102)
14	Translationally controlled tumor protein (TCTP_HUMAN)	Multifunctional protein involved in cell growth, development, apoptosis, regulation of protein synthesis, DNA repair, immune response, malignant transformation, and tumor reversion (103, 104)

**FIG 1:**
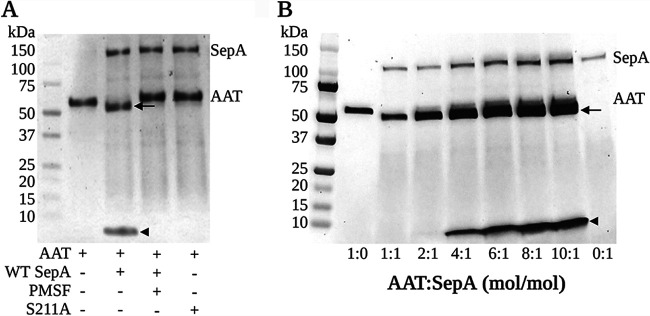
SepA cleaves AAT without generating an inhibitory complex. (A) SDS-PAGE analysis of equimolar reactions between AAT and WT SepA under different conditions. WT SepA proteolytically inactivates AAT by hydrolyzing its Met-358–Ser-359 peptide bond, which results in the production of an ∼50-kDa degraded form of the protein and a 36-amino-acid-long (∼4.2-kDa) C-terminal peptide. The degradation of AAT is no longer observed when PMSF is added to the reaction mixture or when the proteolytically inactive S211A SepA protein is used instead, demonstrating that this effect is dependent on the proteolytic activity of SepA. (B) SepA can cleave increasing concentrations of AAT without the generation of an inhibitory complex. Arrow, 50-kDa cleaved AAT; arrowhead, 4.2-kDa C-terminal peptide. This image was created with BioRender.

10.1128/mBio.02833-21.1 FIG S1 Two-dimensional difference gel electrophoresis (2D-DIGE) reveals SepA cleavage targets. (A) Experimental scheme. (B) 2D-DIGE overlay of samples of human colon incubated with WT SepA and labeled with Cy3 (green spots) or incubated with the inactive S211A protein and labeled with Cy5 (red spots). Twenty-four differences were identified by ImageQuant software (white circles). Protein content was separated with a pH range of approximately 4 to 9 from left to right in the horizontal dimension; the molecular weight range was 10 kDa to 110 kDa from bottom to top in the vertical dimension. (C) Spots 5 and 6 were observed only for the incubation with inactive protein, and spot 24 at a smaller apparent molecular weight was observed only for the active protein. All three spots were identified as alpha-1 antitrypsin (AAT). (D) Edman degradation of bands X and Y resulting from *in vitro* digestion of AAT with SepA (as shown in Fig. 1A). The arrowhead indicates the cleavage site of SepA on AAT. This image was created with BioRender. Download FIG S1, TIF file, 1.1 MB. Copyright © 2021 Meza-Segura et al. 2021 Meza-Segura et al. https://creativecommons.org/licenses/by/4.0/ This content is distributed under the terms of the Creative Commons Attribution 4.0 International license.

### SepA cleaves AAT, escaping the formation of an inhibitory complex.

AAT was prominent among the SepA cleavage targets identified (Table 1), and our interest was piqued because AAT is a serpin family serine protease inhibitor that forms complexes with its target protease only after getting cleaved. Thus, we aimed to investigate the potential role of AAT in inhibiting the activity of SepA. We began by validating the result obtained by 2D-DIGE (Table 1) and performed a biochemical analysis in which AAT was incubated with equimolar concentrations of purified WT or S211A SepA for 1 h at 37°C. As shown in Fig. 1A, AAT is cleaved into two fragments of ∼50 and 4 kDa only when exposed to WT SepA. The addition of a serine protease inhibitor (phenylmethylsulfonyl fluoride [PMSF]) or incubation with a serine-defective SepA (S211A mutant) protein did not result in AAT cleavage (Fig. 1A). These results suggest that the serine protease activity of SepA specifically accounts for the cleavage of AAT.

Additionally, Edman degradation sequence analysis of the AAT-WT SepA reaction products revealed that SepA proteolytically inactivates AAT by hydrolyzing the Met-358–Ser-359 peptide bond of the mature protein, which corresponds to Met-382–Ser-383 in the precursor, which contains a 24-amino-acid signal peptide (Fig. S1D). This peptide bond consists of the P1 to P1′ residues in the reactive center loop (RCL) of AAT (22). The RCL of AAT acts as a bait for serine proteases and forms a covalent bond at the active center once AAT is cleaved, creating an inhibitory complex (23). Once cleaved, a 36-amino-acid (∼4- kDa) peptide is generated from the C-terminal region of AAT (22, 24). Our results based on Edman degradation sequence analysis confirmed that this C-terminal peptide corresponds to the ∼4-kDa residue observed when AAT was incubated with WT SepA (Fig. S1D). Although we did not observe an inhibitory complex between AAT and SepA, we did identify an ∼50-kDa cleavage product of AAT (Fig. 1A, arrow), suggesting that SepA cleaves AAT, releasing the C-terminal peptide of the protein, but escapes the formation of the inhibitory complex. Unlike with SepA, we observed that AAT forms an inhibitory complex with another *Shigella* SPATE, SigA (see Fig. 5A), confirming the inhibitory activity of AAT under the conditions tested.

Previous reports have demonstrated that inhibitory complexes formed between AAT and serine proteases decay with time and are dissociated in alkaline solutions (25, 26). Thus, we tested WT SepA cleavage of AAT through a range of different pHs (pHs 5 to 9) or incubation times (from 0 to 60 min) but were unable to identify an inhibitory complex between AAT and SepA under any of the conditions tested (Fig. S2). Moreover, maximum cleavage of AAT was determined to be within a pH range of 7.4 and 8.2 (Fig. S2A), and unless otherwise stated, a pH of 7.4 was used for further experiments.

10.1128/mBio.02833-21.2 FIG S2 Changes in pH or incubation time do not result in the formation of an inhibitory complex between AAT and WT SepA. (A) Reaction mixtures containing AAT and WT SepA in a 2:1, mol/mol, ratio were prepared in different buffers and incubated for 1 h at 37°C under the following conditions: at pH 5.0 with 0.1 M sodium acetate, at pH 6.2 with 0.1 M MES (morpholineethanesulfonic acid), at pH 6.8 with 0.1 M HEPES, at pH 7.4 with 1× PBS, at pH 8.2 with 50 mM Tris-HCl, and at pH 9.0 with 50 mM Tris-HCl. (B) Equimolar reaction mixtures of AAT and WT SepA were incubated at 37°C for different periods of time. Arrow, 50-kDa degraded AAT; arrowhead, 4.2-kDa C-terminal peptide. This image was created with BioRender. Download FIG S2, TIF file, 0.7 MB. Copyright © 2021 Meza-Segura et al. 2021 Meza-Segura et al. https://creativecommons.org/licenses/by/4.0/ This content is distributed under the terms of the Creative Commons Attribution 4.0 International license.

By incubating SepA with increasing amounts of AAT for 1 h, we demonstrated that even a 10-times-higher concentration of AAT in a molar ratio did not promote the formation of an inhibitory complex (Fig. 1B). Instead, this reaction resulted in a higher production of the 4.2-kDa peptide (arrowhead). Besides, the proteolytic activity of SepA was not affected by the presence of an equimolar concentration of AAT (Fig. S3) but was found to be significantly higher when a 5 (5:1, AAT to SepA)- or 10 (10:1, AAT to SepA)-times-higher molar concentration of AAT was used (Fig. S3). Taken together, these results further indicate that AAT behaves as a substrate rather than as an inhibitor of SepA.

10.1128/mBio.02833-21.3 FIG S3 AAT does not inhibit WT SepA. The proteolytic activity of SepA was determined by quantifying the degradation of the Suc-AAPF-pNA synthetic peptide. Equimolar concentrations of AAT and WT SepA did not affect the catalytic activity of the protease. Higher concentrations of AAT (5:1 and 10:1) resulted in a significant increase in the proteolytic activity of WT SepA. Asterisks denote *P* values of <0.05, as determined by the 2-tailed nonparametric Mann-Whitney U test. Data are means ± SE from six replicates, and results are representative of experiments with PMNs from different donors. This image was created with BioRender. Download FIG S3, TIF file, 0.2 MB. Copyright © 2021 Meza-Segura et al. 2021 Meza-Segura et al. https://creativecommons.org/licenses/by/4.0/ This content is distributed under the terms of the Creative Commons Attribution 4.0 International license.

### SepA-cleaved AAT induces neutrophil migration.

**FIG 2:**
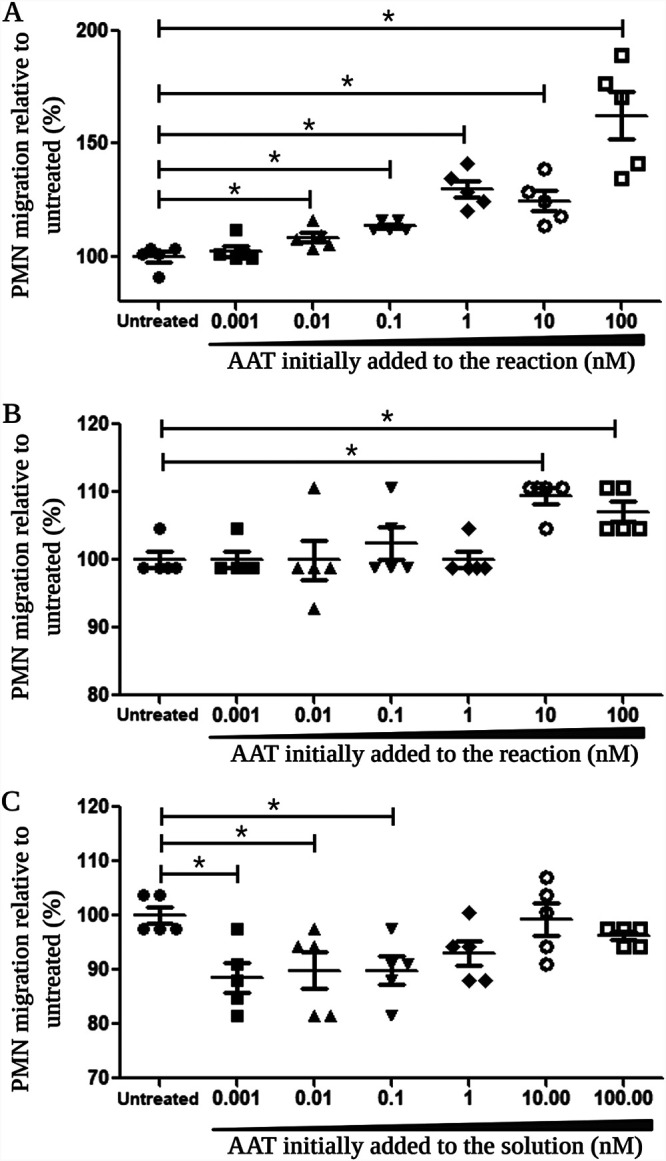
The products of the reaction between AAT and WT SepA stimulate the migration of PMNs. A reaction mixture containing 10 μM AAT and 1 μM WT SepA was incubated at 37°C for 4 h, and the reaction was stopped by addition of a 10-fold molar excess of PMSF. Logarithmic dilutions of this reaction mixture, corresponding to concentrations between 0.001 and 1.00 nM AAT initially added to the reaction mixture, were tested for their chemotactic activity. (A) AAT-WT SepA reaction products induced a significant increase in PMN migration through acellular-collagen-coated transwells in concentrations equivalent to 0.01 to 100 nM AAT. (B) The transepithelial migration of PMNs through polarized T84 cell monolayers was significantly stimulated by the AAT-WT SepA reaction products in concentrations equivalent to 10 to 100 nM AAT. (C) Low concentrations of AAT (0.001 to 0.1 nM) in the absence of WT SepA significantly inhibited the transepithelial migration of PMNs in the polarized T84 cell model in comparison to that of untreated controls. Asterisks indicate a significant difference in PMN migration from that of the untreated controls (*P* < 0.05, 2-tailed nonparametric Mann-Whitney U test). Data are means ± standard errors (SE) from five replicates for all conditions tested, and results are representative of experiments with PMNs from different donors. This image was created with BioRender.

Prior studies determined that the 4.2-kDa AAT C-terminal peptide (cAAT) generated from the cleavage of AAT by neutrophil elastase (NE) stimulates the migration of neutrophils (24). Since the same 4.2-kDa cAAT peptide was obtained by SepA cleavage of AAT, we reasoned that this product may have the same effect on polymorphonuclear leukocyte (PMN) migration during S. flexneri infection. To test this notion, we began by assessing the chemotactic activity of the AAT-WT SepA reaction products in our acellular transwell migration model system. Briefly, freshly purified PMNs were applied to the top compartment of acellular collagen-coated transwells, and serial dilutions of the AAT-WT SepA reaction products were added to the bottom of each well. Given that we used the whole product of this reaction, results are presented relative to the initial concentration of AAT in the reaction mixture. As expected, concentrations higher than 0.01 nM AAT induced a significant increase in PMN migration compared to that in untreated media, reaching a maximal migration response at a 100 nM concentration (1.6-fold higher than that of untreated media) (Fig. 2A).

Next, we used our well-characterized *in vitro* PMN transepithelial migration system with polarized T84 cell monolayers to determine the extent to which the products of the AAT-WT SepA reaction were able to stimulate the transepithelial migration of PMNs (27, 28). Under these conditions, we observed chemotactic activity within the AAT-WT SepA reaction products that resulted in a significant induction of PMN migration (10 to 100 nM AAT) (Fig. 2B). This increase in PMN migration was not due to cytotoxic effects, as lactate dehydrogenase (LDH) assays revealed no significant cellular damage to either PMNs or T84 cell monolayers following treatment with AAT-WT SepA products in the range of concentrations used (Fig. S4). In contrast, different concentrations of AAT (0.001 to 0.1 nM) in the absence of WT SepA showed a significant reduction in PMN migration through T84 cell monolayers compared to that in untreated media (Fig. 2C). Hence, such results indicate that the chemotactic activity of AAT is generated only after it becomes cleaved by SepA.

10.1128/mBio.02833-21.4 FIG S4 AAT-WT SepA reaction products are not toxic for T84 cells or PMNs. The release of LDH from T84 cells (A) and human PMNs (B) was quantified after incubation for 3 h at 37°C in the presence of different concentrations of the products of the AAT-WT SepA reaction. AAT-WT SepA reaction products were obtained from a 10 μM AAT and 1 μM WT SepA solution incubated at 37°C for 4 h, and the reaction was stopped by adding a 10-fold molar excess of PMSF. Results are presented relative to the initial concentration of AAT in the reaction mixture. Data are means ± SE from 8 replicates for all conditions tested, and results are representative of experiments with PMNs from different donors. This image was created with BioRender. Download FIG S4, TIF file, 0.3 MB. Copyright © 2021 Meza-Segura et al. 2021 Meza-Segura et al. https://creativecommons.org/licenses/by/4.0/ This content is distributed under the terms of the Creative Commons Attribution 4.0 International license.

### AAT enhances PMN migration and bacterial invasion during S. flexneri infection.

**FIG 3:**
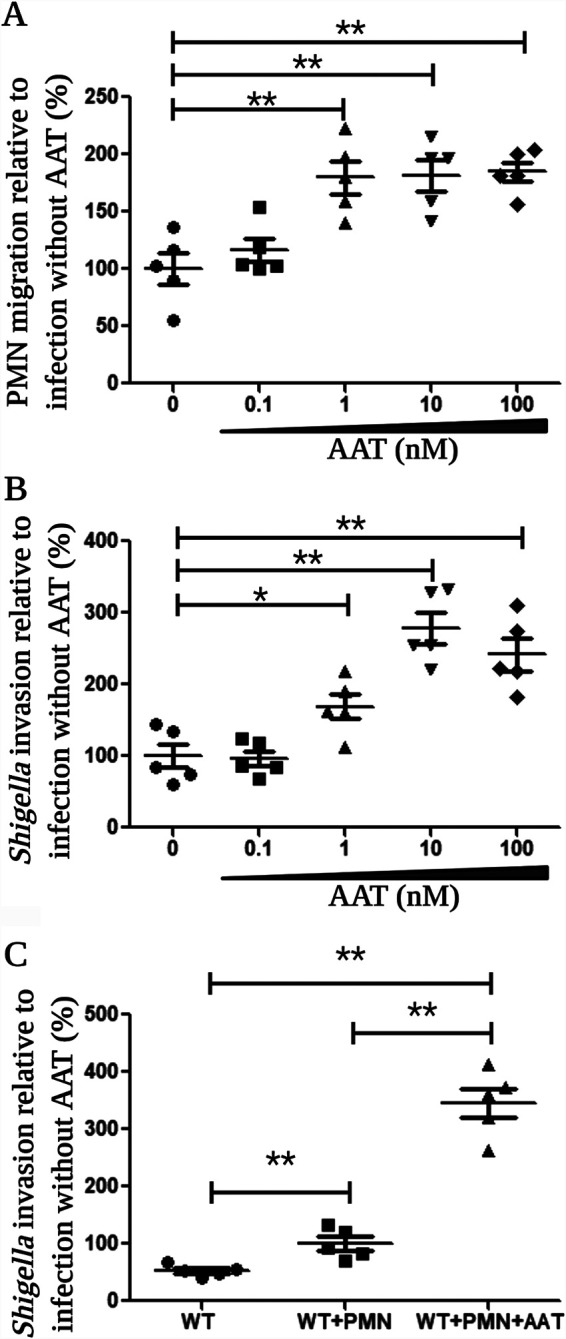
The presence of AAT during *Shigella* infection facilitates PMN migration and enhances the invasion of epithelial cells. (A and B) AAT in concentrations between 1 and 100 nM significantly increased PMN migration (A) and bacterial invasion (B) in comparison to those in cells infected in the absence of AAT. (C) The addition of PMNs and 10 nM AAT during *Shigella* infection led to a 6.5-fold increase in the number of bacteria invading epithelial cells. In comparison, the addition of PMNs alone doubled the number of invading bacteria. The data are expressed as means ± SE from five replicates for all conditions tested. Asterisks denote *P* values of <0.05 (*) and <0.01 (**) as determined by a 2-tailed nonparametric Mann-Whitney U test. This image was created with BioRender.

Since the AAT-SepA reaction products promoted the migration of PMN across cell monolayers, we inferred that the degradation of luminal AAT by SepA might facilitate S. flexneri invasion by stimulating the migration of PMN across the intestinal epithelium and, as a consequence, that it perturbs the epithelial barrier, making it more permissive to breaches, as previously suggested (11). To examine this possibility, we standardized an infection system adapted from the model reported by Perdomo et al. (11) that enables simultaneous quantification of both PMN migration and bacterial invasion. For this model, we performed an “inverse” measurement of PMN migration by quantifying those PMNs that remained in the basolateral compartment (and subtracted this number from the initial amount of PMNs added to the system). The inverse measurement of PMN migration was validated by establishing that both the traditional and the inverse detection of migrating PMNs showed similar outcomes when epithelial cells were infected with WT *Shigella* or with the avirulent BS103 strains (Fig. S5; described in Materials and Methods). Thus, implementing the “combined PMN migration and *Shigella* invasion” model, we observed that following apical infection of T84 monolayers with WT S. flexneri in the presence of AAT at concentrations between 1 and 100 nM, both PMN transepithelial migration (Fig. 3A) and bacterial invasion (Fig. 3B) significantly increased. The highest level of invading S. flexneri bacteria was achieved at a 10 nM concentration of AAT (Fig. 3B), and it was nearly three times higher than the number of bacteria recovered in the absence of AAT.

10.1128/mBio.02833-21.5 FIG S5 Quantification of PMNs in the apical and basolateral sides of epithelial cells. T84 cell monolayers were infected with WT and BS103 *Shigella* strains for 3 h. Cells were washed three times with HBSS+ and placed in fresh medium. PMNs were added to the basolateral side and incubated for 3 h at 37°C. PMNs that migrated to the apical side, as well as the ones that remained in the basolateral side, were collected, lysed, and quantified via a colorimetric peroxidase assay. The results were used to determine and compare the traditional (A) and inverse (B) measurements of PMN migration, demonstrating that both methods show similar trends. Asterisks denote *P* values of <0.05, as determined with the 2-tailed nonparametric Mann-Whitney U test. The data are expressed as means ± SE from four replicates, and results are representative of experiments with PMNs from different donors. This image was created with BioRender. Download FIG S5, TIF file, 0.2 MB. Copyright © 2021 Meza-Segura et al. 2021 Meza-Segura et al. https://creativecommons.org/licenses/by/4.0/ This content is distributed under the terms of the Creative Commons Attribution 4.0 International license.

To control for the effect of PMNs on S. flexneri invasion, we also performed experiments in the absence of PMNs. As shown in Fig. 3C, our results corroborated the results of Perdomo et al., showing that bacterial invasion was enhanced when PMNs were added to the system (WT = 52.93 ± 4.33 versus WT plus PMNs = 100 ± 11.69%, *P* = 0.0079, as determined by the Mann-Whitney U test [MWUT]). Moreover, when PMNs and a 10 nM concentration of AAT were present during S. flexneri infection, we observed a 6.5-fold increase in the number of bacteria invading epithelial cells (WT = 52.93 ± 4.33 versus WT plus PMNs plus AAT = 346 ± 25.48%, *P* = 0.0079, MWUT). On the other hand, when cells were infected in the presence of 100 nM *N*-formyl-methionyl-leucyl-phenylalanine (fMLP), despite the high increase in migration stimulated by this chemoattractant, there was only a mild and not significant increase in bacterial invasion (Fig. S6). Thus, despite the modest level of PMN migration induced by the presence of AAT, it is sufficient and appropriately tuned to facilitate *Shigella* invasion.

10.1128/mBio.02833-21.6 FIG S6 fMLP significantly increased PMN migration but not *Shigella* invasion. (A) The presence of 100 nM fMLP significantly increased the infiltration of PMNs induced by *Shigella* infection. This increase was significantly higher than the one observed with 10 nM AAT and the one observed with fMLP in uninfected cells. (B) Bacterial invasion is enhanced in the presence of 10 nM AAT but not with fMLP. Asterisks denote *P* values of <0.05 (*) and <0.01 (**), as determined by a 2-tailed nonparametric Mann-Whitney U test. The data are expressed as means ± SE from five replicates, and results are representative of experiments with PMNs from different donors. This image was created with BioRender. Download FIG S6, TIF file, 0.2 MB. Copyright © 2021 Meza-Segura et al. 2021 Meza-Segura et al. https://creativecommons.org/licenses/by/4.0/ This content is distributed under the terms of the Creative Commons Attribution 4.0 International license.

### SepA is sufficient to enhance neutrophil migration and *Shigella* invasion in the presence of AAT.

**FIG 4:**
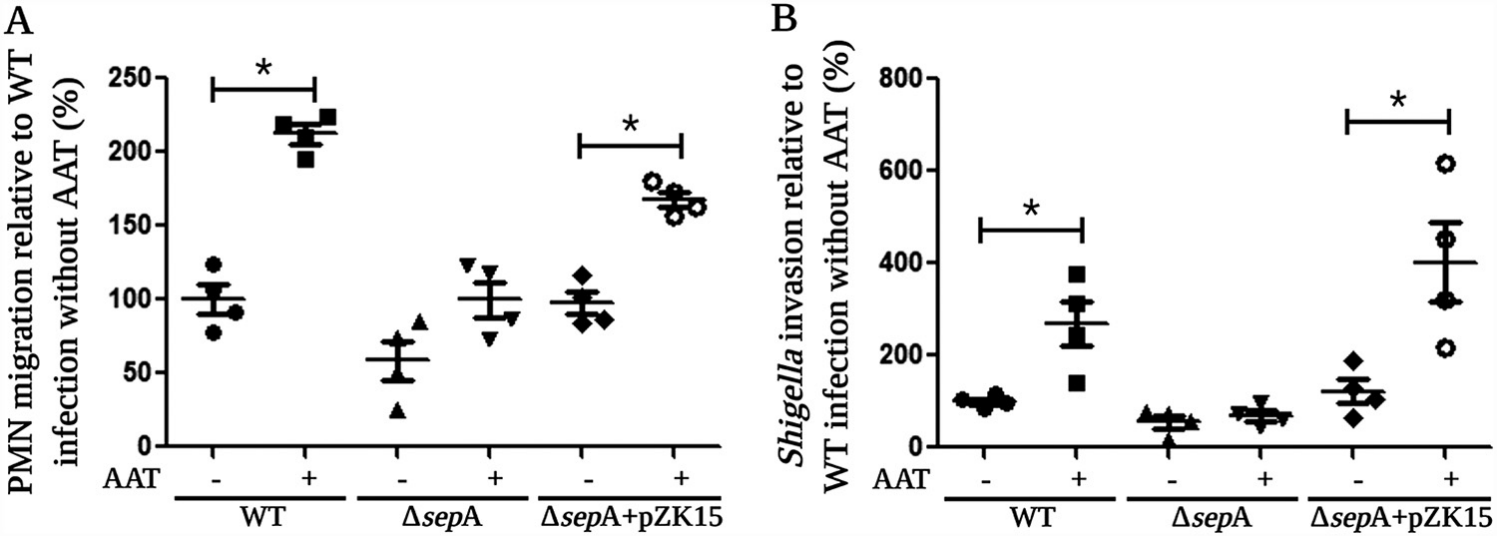
SepA is essential to enhance neutrophil migration and *Shigella* invasion in the presence of AAT. The significant increases in PMN migration (A) and bacterial invasion (B) stimulated by the presence of AAT are dependent on the expression of SepA, with both effects getting abrogated when cells were infected with a *sepA* mutant strain (Δ*sepA*) and restored when the mutant was complemented with a plasmid to express *sepA* (pZK15). Asterisks denote *P* values of <0.05 as determined by a 2-tailed nonparametric Mann-Whitney U test. Data are means ± SE from four replicates, and results are representative of experiments with PMNs from different donors. This image was created with BioRender.

To further assess the role of SepA during S. flexneri infection in the presence of AAT, we used our combined PMN migration and *Shigella* invasion model to examine an isogenic mutant strain lacking the gene encoding SepA (Δ*sepA*), as well as a Δ*sepA* mutant complemented with an inducible plasmid to express *sepA* (pZK15). Following infection with these strains, we observed that the presence of AAT induced a significant increase in PMN migration and bacterial invasion only in those monolayers infected with *sepA*-harboring strains (Fig. 4A and B). Given that no significant changes were observed when epithelial cells were infected with a Δ*sepA* mutant in the presence or absence of AAT, these results demonstrate that SepA is essential for the increase in PMN migration and bacterial invasion induced by AAT during *Shigella* infection. Yet, we did note a consistently mild but not significant rise in PMN migration when cell monolayers were infected with the Δ*sepA* strain in the presence of AAT, leaving open the possibility that other serine proteases secreted by *Shigella* might play a supportive role by degrading AAT and increasing the production of cAAT. Unexpectedly, by using the combined PMN migration and *Shigella* invasion model in the absence of AAT, we were not able to detect a significant reduction in PMN migration when epithelial monolayers were infected with the *sepA* mutant in comparison to that of cells infected with WT S. flexneri (WT = 100 ± 9.84% versus Δ*sepA *mutant = 58.7 ± 13.3%, *P* = 0.0571, MWUT), as previously reported (12). However, such differences became evident and significant when AAT was present (WT plus AAT = 212.1 ± 6.29% versus Δ*sepA* mutant plus AAT = 99.98 ± 12.09%, *P* = 0.0286, MWUT). Moreover, infection with the Δ*sepA* strain resulted in reduced bacterial invasion both in the absence (WT = 100 ± 6.58% versus Δ*sepA *mutant = 55.56 ± 13.53%, *P* = 0.0286, MWUT) and in the presence (WT = 268.6 ± 49.93% versus Δ*sepA *mutant = 68.52 ± 10.2%, *P* = 0.0286, MWUT) of AAT.

**FIG 5:**
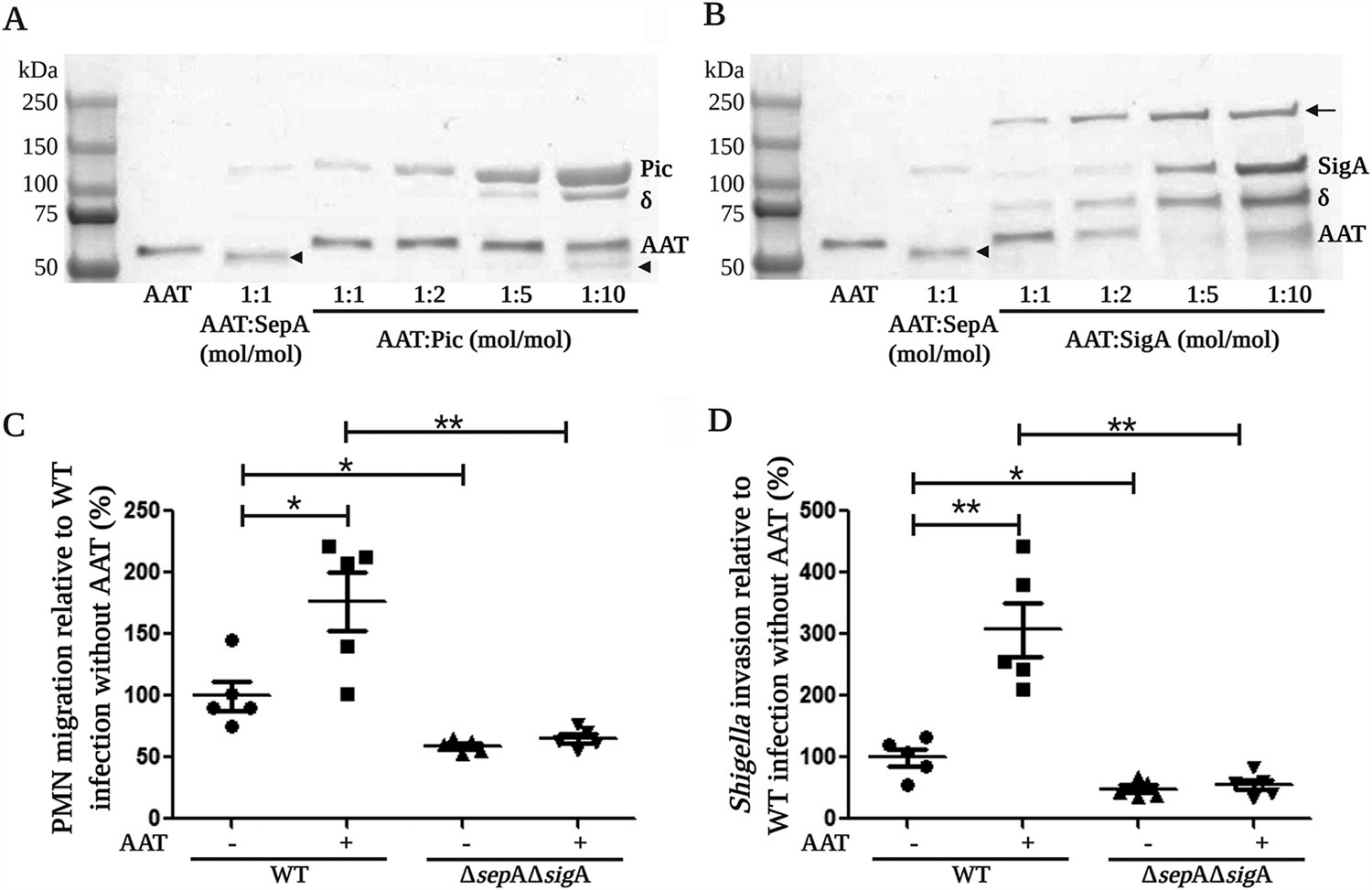
SigA forms an inhibitory complex with AAT and participates in the induction of PMN migration. (A) Pic cleaved a small amount of AAT only when it was added at a 10-times-higher concentration (1:10), but no formation of an inhibitory complex was detected. (B) SigA and AAT formed a complex that became more evident at higher concentrations of SigA. (C and D) Infection of epithelial cells with a SepA and SigA double mutant (Δ*sepA* Δ*sigA*) completely abrogated the increase in PMN migration stimulated by the presence of AAT (C) and resulted in a lower number of bacteria invading epithelial cells (D). Asterisks denote *P* values of <0.05 (*) and <0.01 (**) as determined by a 2-tailed nonparametric Mann-Whitney U test. (C and D) Data are means ± SE from five replicates, and results are representative of experiments with PMNs from different donors. δ, Pic and SigA breakdown products; arrowhead, 50-kDa cleaved AAT; arrow, AAT-SigA inhibitory complex. This image was created with BioRender.

Next, we reasoned that besides generating a PMN chemoattractant, the degradation of AAT might hinder the inhibition of other serine proteases important for *Shigella* pathogenesis. Thus, we analyzed the effect of AAT on the proteolytic activities of Pic and SigA, as well as its potential to form inhibitory complexes with both proteases. For Pic, we did not detect the formation of an inhibitory complex with AAT (Fig. 5A). However, when AAT was exposed to a 10-fold molar excess of Pic (1:10 AAT to Pic, mol/mol) we observed a small amount of cleaved protein (arrowhead) (Fig. 5A). Of note, a band of ∼100 kDa (δ) was detected at high concentrations of Pic, which corresponds to a breakdown product of Pic, an observation that has been reported for other SPATES (29–31). Moreover, no significant inhibition of Pic’s proteolytic activity was detected in the presence of increasing concentrations of AAT (Fig. S7A). These results suggest that as with SepA, Pic can cleave AAT and escape the formation of an inhibitory complex. However, unlike with SepA, Pic-mediated cleavage of AAT appears to be a rather inefficient process. Concerning SigA, we observed the formation of an ∼150-kDa inhibitory complex when the protease was incubated with an equimolar concentration of AAT (Fig. 5B, arrowhead), which became more evident by increasing the concentration of SigA. Similarly to what was observed for Pic, an ∼75-kDa breakdown product of SigA (δ) was also detected. What is more, an equimolar concentration of AAT was sufficient to completely inhibit SigA’s proteolytic activity (Fig. S7B). All together, these results indicate that SigA cleaves AAT at its P1–P1′ peptide bond and forms a stable complex with the inhibitor; thus, besides SepA, SigA may participate to a minor extent in the generation of cAAT.

10.1128/mBio.02833-21.7 FIG S7 AAT inhibits SigA but not Pic. The proteolytic activities of Pic and SigA were determined by quantifying the degradation of the Suc-AAP-Abu-pNA synthetic peptide. (A) The proteolytic activity of Pic was not affected by the presence of AAT. (B) An equimolar concentration of AAT was enough to completely inhibit the proteolytic activity of SigA. Asterisks denote *P* values of <0.05, as determined by a 2-tailed nonparametric Mann-Whitney U test. Data are means ± SE from four replicates, and results are representative of experiments with PMNs from different donors. This image was created with BioRender. Download FIG S7, TIF file, 0.3 MB. Copyright © 2021 Meza-Segura et al. 2021 Meza-Segura et al. https://creativecommons.org/licenses/by/4.0/ This content is distributed under the terms of the Creative Commons Attribution 4.0 International license.

Therefore, we evaluated the effect of AAT during an infection with a double mutant of *Shigella* lacking the genes encoding SepA and SigA (Δ*sepA* Δ*sigA* mutant), both of which are relevant for AAT cleavage. By using the Δ*sepA* Δ*sigA* strain, we observed that the increases in both PMN migration (Fig. 5C) and bacterial invasion (Fig. 5D) induced by AAT were completely abrogated. Moreover, in the absence of AAT, infection with the S. flexneri Δ*sepA* Δ*sigA* double mutant resulted in a significant reduction in PMN migration and bacterial invasion compared to that of monolayers infected with WT S. flexneri. This finding indicates that both serine proteases, SepA and SigA, also participate in those two crucial events of S. flexneri pathogenesis in an AAT-independent manner.

## DISCUSSION

Previously, we reported that SepA is crucial for *Shigella* pathogenesis through its ability to disrupt the intestinal epithelial barrier, which facilitates subsequent bacterial transit to the basolateral pole (12). While we demonstrated that this effect was orchestrated by the activation of cofilin via LIMK1 downregulation, SepA was not able to directly degrade LIMK1 *in vitro* (12). Therefore, in an attempt to more directly determine the targets of SepA’s proteolytic activity, we incubated a sample of freshly homogenized human colon in the presence of WT SepA or an inactive mutant of this protease. This analysis identified 18 different protein targets of SepA (Table 1), leading to the inference that SepA interacts with a variety of substrates. Given that SepA is the most abundant protein secreted by S. flexneri (at least *in vitro* [14]), and in recognition of the diverse niches that *Shigella* encounters within the intestinal microenvironment, this finding is perhaps not surprising and is consistent with what has been described for other SPATES (13, 21).

The degradation of SepA targets may play an important role in the colonization of the colonic epithelium by facilitating different stages of *Shigella* pathogenesis. For instance, the cleavage of galectin-4, a protein suggested to help maintain the integrity of the epithelial barrier (32, 33), may facilitate the opening of intercellular junctions and promote the basolateral translocation of *Shigella*. The degradation of proteins involved in cytoskeleton dynamics (such as IQGAP1, gamma-actin, and alpha-actinin), may be of relevance for the basolateral invasion of epithelial cells and the invasion of neighboring cells (34, 35). Moreover, the degradation of an anterior gradient protein 2 homolog may result in a reduction of intestinal mucin production (36). Of note, even though we found cofilin to be a cleavage target of SepA in this work (spot 20 in Table 1), we failed to observe such a cleavage event, as described in our prior study (12). A possible explanation for this apparent discrepancy may be the distinct nature of the experimental setups; the current study employed an extract from homogenized human colon and purified SepA, whereas our prior study used human intestinal epithelial cell monolayers infected with various S. flexneri strains. While beyond the scope of the current report, future studies to address this difference are planned.

Regardless, the central finding from this study is that we identified AAT to be a key target of SepA. AAT is a major circulating serine proteinase inhibitor in humans (37). The inhibitory function of AAT depends on its RCL, which acts as a bait for serine proteases (23). After binding to the RCL of AAT, serine proteases hydrolyze the P1–P1′ peptide bond (M358–S359), forming an acyl-intermediate bond with the backbone carbon of the P1 residue (38). The cleavage of the AAT RCL gives rise to a large-scale structural rearrangement from a stressed to a relaxed conformation, which distorts the protease’s catalytic center, leading to formation of the classic tightly bound serpin inhibitory complex (39).

We found that SepA was able to cleave AAT without the formation of an inhibitory complex, thus evading the potent inhibitory activity characteristic of serpins. This activity is unusual but not unprecedented. The V8 serine protease secreted by Staphylococcus aureus (40) is able to cleave AAT without forming an inhibitory complex. However, while SepA hydrolyzes the P1–P1′ peptide bond in the RCL of AAT, the V8 protease cleaves the Glu354–Ala355 peptide bond, similar to what has been described for cysteine proteases (40, 41). An analogous effect has been described for the degradation of another serpin, alpha-1 antichymotrypsin (ACT), by neutrophil elastase, showing that ACT behaves as a substrate rather than an inhibitor of the enzyme (42, 43). Finally, the SPATE EspPα from enterohemorrhagic E. coli also has been shown to cleave and inactivate AAT without the formation of an inhibitory complex (31). As with SepA, cleavage occurs in the AAT RCL (31).

We observed that high concentrations of AAT induced a significant increase in the proteolytic activity of SepA. A similar phenomenon, termed “activation by an excess of the substrate,” has been reported for other serine proteases, and is thought to result from the binding of a second substrate molecule to a regulatory site of the enzyme (44–48). We, therefore, speculate that the activation of SepA by an excess of AAT may be physiologically relevant during the earlier stages of S. flexneri infection when the amount of synthesized SepA would be very low compared to the concentration of AAT in the intestinal lumen.

AAT is known to exert dual effects depending on its structure (49). In its native form, AAT has anti-inflammatory effects, as it has been observed in the lungs of patients with AAT deficiency after AAT replenishment or in patients with cystic fibrosis and pneumonia after exogenous supplementation with AAT (50–52). Furthermore, *in vitro* AAT can reduce the production of proinflammatory cytokines in monocytes and inhibit the migration of PMNs (53–58). However, when AAT is hydrolyzed by the action of serine proteases, a 36-amino-acid peptide is generated from its C-terminal domain (cAAT). This product can stimulate PMN chemotaxis, adhesion, degranulation, and superoxide generation (24, 59, 60). In agreement with these observations, we demonstrate that the degradation products of AAT (generated by the proteolytic activity of SepA) induce a dose-dependent increase in PMN migration through acellular collagen-coated transwells. We also observed that the AAT-SepA reaction products stimulated the transmigration of PMNs from the basolateral to the apical side of epithelial cell monolayers. Maximal induction of neutrophil transepithelial migration was detected at a 10 nM concentration of AAT (initially added to the reaction mixture), which is comparable to the concentration of AAT necessary to promote a maximal chemotactic effect following its degradation by human neutrophil or mouse macrophage elastase (24, 59).

AAT is also an acute-phase protein that is commonly used to determine the leaking of serum proteins into the gut in patients suffering gastrointestinal disorders, including inflammatory bowel diseases or intestinal infections (61). In line with this observation, shigellosis is associated with high concentrations of AAT in the stool (average of 10.9 mg/g [dry weight] of stool) in comparison to what has been reported for healthy subjects (<2 mg/g [dry weight]) (62, 63). This rise in stool AAT is associated with the presence of erythrocytes and is inversely correlated with serum protein concentrations, demonstrating the leakage of plasma proteins in the gut (63–65). Since one of the clinical features of shigellosis is the presence of high numbers of PMNs in the colonic lamina propria and surface epithelium, AAT may be important to protect the intestinal mucosa from the action of PMN serine proteases during *Shigella* infection (66–69).

Despite their being the first line of defense against bacterial infections, the recruitment of PMNs to the intestinal lumen can be advantageous for *Shigella* pathogenesis at early stages of infection by disrupting the intestinal epithelial barrier and loosening the intercellular junctions (10, 11), thus allowing *Shigella* to travel to the basolateral pole, from which it can efficiently invade the epithelium. On the balance of our results, we posit that this effect is further facilitated by the degradation of luminal AAT due to SepA’s proteolytic activity, the sum of which produces a cAAT peptide chemoattractant that enhances PMN migration and the subsequent invasion of epithelial cells at the basolateral surface.

A potential limitation of our *in vitro* migration invasion system is that it does not allow us to investigate whether the degradation products of the AAT-SepA reaction are sufficient to induce the initial recruitment of PMNs from the bloodstream to the submucosa. Nevertheless, our data support the concept that cAAT is involved in the generation of a PMN chemoattractant gradient that ultimately directs PMNs to cross the intestinal epithelium from the submucosa to the intestinal lumen, similar to what has been described for another PMN chemoattractant, hepoxilin A_3_ (28, 70, 71). In this context, we posit that there is a temporal cascade of chemoattractants that support the movement of neutrophils across the intestinal epithelium. Therefore, the participation of cAAT would be preceded by the basolateral secretion of interleukin-8 (IL-8), which drives the recruitment of neutrophils into the submucosa. There is a significant increase in the synthesis and secretion of IL-8 in the intestinal epithelium during infection with S. flexneri, and it is also documented that this occurs early after infection (72, 73). In addition, human biopsy specimens collected from patients with shigellosis showed that IL-8 remains high in the acute and convalescent stages of the disease (74). It also appears that multiple T3SS effectors contribute to this process by activating the mitogen-activated protein kinase (MAPK)/extracellular signal-regulated kinase (ERK) signaling pathway and promoting the secretion of IL-8, including OspB, OspC1, and OspZ (75–78).

Curiously, in comparing the effect of AAT during S. flexneri infection to the action of the prototypical PMN chemoattractant, fMLP, we observed that despite the higher induction of PMN migration over AAT, there was an insignificant increase in bacterial invasion. This result implies that while the induction of PMN migration contributes to pathogenesis, the recruitment of high numbers of PMNs may affect the progression of the infection. Hence, we favor the hypothesis that the more modest chemotactic effect exhibited by the AAT-SepA reaction products better enables bacterial invasion. In other words, a less potent PMN chemoattractant may dampen inflammation sufficiently to allow for S. flexneri invasion of the epithelium, which would be most critical at early stages of infection. Eventually, however, a more robust inflammatory response presumably induced by HXA_3_ (70) likely leads to *Shigella* clearance, since PMNs eliminate the infection within 5 to 7 days in healthy individuals (67). Of note, at this later stage of the infection, the concentration of stool AAT also shows a significant reduction (2.2 mg/g), most likely due to healing of the epithelial barrier (63).

In a previous study, we demonstrated that SepA facilitates the migration of PMNs and the invasion of epithelial cells by S. flexneri in the absence of AAT (12). In the current work, we also detected a pronounced trend in the reduction (*P* = 0.0571) of PMN migration after infecting T84 cells in the absence of AAT with the Δ*sepA* strain compared to WT *Shigella*. We believe that this trend approached but did not reach significance because of the modifications made to the infection model. For instance, since PMNs were present at the time of the infection in the combined PMN migration and *Shigella* invasion model, an inverse measurement of PMN migration was performed to avoid the interference of bacteria in the solution used for quantification. Unlike with the PMN migration results, we did observe a significant reduction in bacterial invasion when T84 cell monolayers were infected with the Δ*sepA* strain in comparison to that with WT *Shigella*. However, further optimization of the model system is required to rule out the possibility that SepA has AAT-dependent and AAT-independent effects in PMN transmigration and bacterial invasion.

By cleaving AAT without forming an inhibitory complex, SepA may also impact other serine proteases essential for *Shigella* pathogenesis. In this regard, our results revealed that SigA is inhibited by an equimolar concentration of AAT. This serine protease has been described to mediate the accumulation of fluid in rabbit ileal loops and to elicit a cytopathic effect in nonconfluent epithelial cells by degrading fodrin (79, 80). These effects are similar to those reported for Pet and EspC, two other SPATES secreted by enteroaggregative and enteropathogenic Escherichia coli strains, respectively (13). Despite the presence of high anti-SigA titers detected following *Shigella* infection, the precise role of this protease remains unknown (80). Still, it is possible that in addition to SigA, other serine proteases crucial for *Shigella* pathogenesis may become inactivated by AAT, further underscoring the importance of SepA, and future studies will investigate this notion.

**FIG 6:**
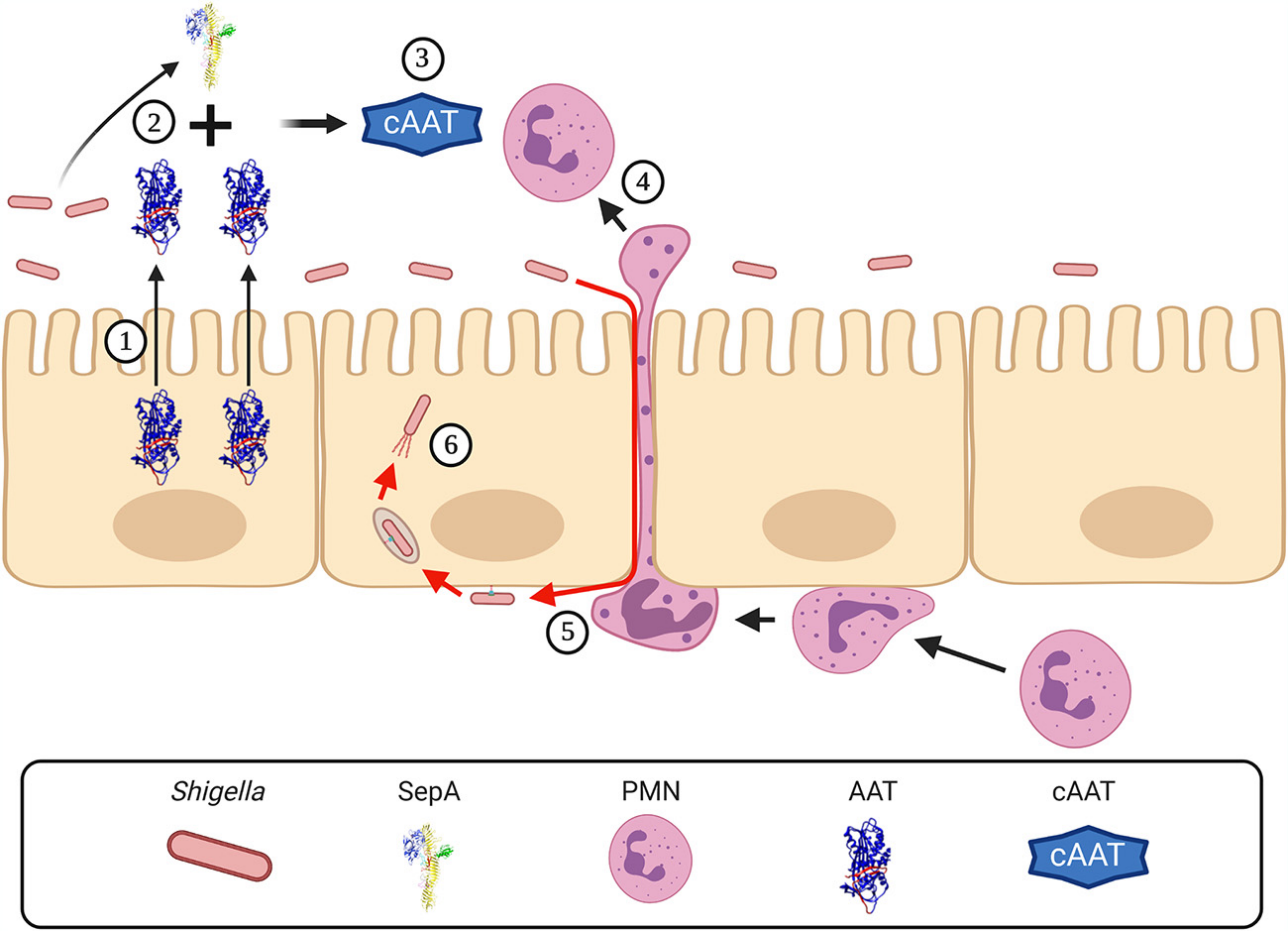
SepA facilitates *Shigella* invasion by degrading AAT and inducing the migration of neutrophils. (1) AAT is secreted to the intestinal lumen by epithelial cells. During *Shigella* infection, SepA degrades AAT (2) and produces a 4.2-kDa C-terminal peptide (cAAT) (3) that stimulates the recruitment of neutrophils to the intestinal lumen (4). While migrating, neutrophils alter the integrity of the epithelial barrier, facilitating the translocation of *Shigella* to the basolateral pole (5) and the subsequent invasion of the epithelial cells (6). This image was created with BioRender.

In sum, we described for the first time that AAT is a key target of SepA. Functionally, by cleaving AAT, SepA releases a 4.2-kDa peptide that promotes neutrophil migration, which in turn disrupts the intestinal epithelial barrier to enable *Shigella* invasion (Fig. 6). These results are consistent with the hypothesis that PMN migration facilitates *Shigella* invasion by loosening the intercellular junctions of intestinal epithelial cells, which allows the bacteria to travel to the basolateral pole (11).

## MATERIALS AND METHODS

### Bacterial strains.

A total of four plasmids and 10 S. flexneri strains were used in this study (see Table S1 in the supplemental material). Both the 2457T Δ*sepA* and 2457T Δ*sepA* plus pZK15 strains were obtained from the Maurelli laboratory (14). Plasmid pS211A, encoding a catalytically inactive serine protease, SepA, with a serine 211-to-alanine point mutation (S211A), was previously described (12). The lambda red recombination system was used as previously described (81) to generate Δ*sepA* and BS103 derivative mutants lacking the gene encoding SigA (*sigA*), without affecting any upstream or downstream genes. The BS103 Δ*sigA* strain was further modified to delete the gene encoding Pic (*pic*) to generate the double mutant strain BS103 Δ*pic* Δ*sigA*. PCR primers used to amplify the kanamycin resistance cassette for lambda red recombination and to identify each S. flexneri derivative are shown in Table S2. Bacterial cultures were stored in 20% glycerol at −80°C. Bacteria were streaked onto tryptic soy agar plates with 0.2% Congo red, and plates were incubated at 37°C for 18 h. For infections, one colony was selected from a fresh plate and was incubated overnight in tryptic soy broth, with ampicillin (100 μg/ml) and IPTG (isopropyl-β-d-thiogalactopyranoside; 0.5 mM) when required. Approximately 14 h later, bacterial cultures were diluted 1:100 in fresh tryptic soy broth and incubated for 2 1/2 h, again, with ampicillin and IPTG when required. Subsequently, bacteria were prepared by washing them once with Hanks’ balanced salt solution (HBSS+, 14025092; Thermo Fisher Scientific) and resuspending them in the same buffer. It should be noted that we detected the expression of SepA in the Δ*sepA* plus pZK15 strain even in the absence of IPTG. SepA expression might be driven by the *sepA* promoter located within the 259-nucleotide sequence upstream of *sepA*’s start codon, which was included in the pZK15 plasmid when *sepA* was originally cloned.

10.1128/mBio.02833-21.8 TABLE S1 Strains and plasmids used in this work. Download Table S1, DOCX file, 0.01 MB. Copyright © 2021 Meza-Segura et al. 2021 Meza-Segura et al. https://creativecommons.org/licenses/by/4.0/ This content is distributed under the terms of the Creative Commons Attribution 4.0 International license.

10.1128/mBio.02833-21.9 TABLE S2 Primers used in this work. Download Table S2, DOCX file, 0.01 MB. Copyright © 2021 Meza-Segura et al. 2021 Meza-Segura et al. https://creativecommons.org/licenses/by/4.0/ This content is distributed under the terms of the Creative Commons Attribution 4.0 International license.

### Pic, SepA, and SigA protein purification.

Genes encoding full-length Pic and SigA were amplified using the Expand long-template PCR system from Roche (catalog no. 11681842001). PCR amplicons containing *pic* and *sigA* were cloned into pUC19 to generate pPic1 and pSigA3, respectively. BS103 Δ*pic* Δ*sigA* was transformed with plasmids encoding SepA (pZK15), inactive SepA (pS211A), Pic (pPic1), or SigA (pSigA3). Each strain was grown in tryptic soy broth at 37°C with shaking (200 rpm) to an optical density at 600 nm (OD_600_) of 0.6. Then, bacteria were induced with IPTG (0.5 mM) for 6 h at 30°C with shaking. Bacterial cultures were centrifuged at 5,000 × *g* for 20 min at 4°C. Supernatants were collected, filtered through a 0.2-μm polyethersulfone filter (ThermoFisher; 569-0020), and concentrated 1,000-fold using an Amicon stirred-cell 400-ml filter unit (Millipore; UFSC40001) with a 30-kDa-cutoff ultrafiltration disc (Millipore; PLTK07610). The concentrated supernatant was then loaded onto a HiLoad 16/600 Superdex 200-pg gel filtration column (Cytiva; 28989335) previously equilibrated with phosphate-buffered saline (PBS). Fractions corresponding to the passenger domain of each protein were concentrated to 10 mg/ml.

### Tissue homogenization and 2D-DIGE.

In order to discover SepA protein cleavage targets, a human colon tissue homogenate was prepared and incubated with SepA and cleavage products were detected by two-dimensional difference gel electrophoresis (2D-DIGE) and tandem mass spectrometry (MS/MS). Specifically, a section of fresh healthy human colon measuring approximately 2 cm by 2 cm was obtained as a discarded surgical by-product from the University of Massachusetts Medical School Cancer Center Tissue Bank (internal review board [IRB] docket no. H1173). This tissue was subjected to homogenization by 20 strokes using a Dounce tissue homogenizer, on ice. The sheared tissue was centrifuged at 15,000 × *g* for 30 min at 4°C, and the supernatant was subsequently incubated either with purified wild-type (WT) SepA or with the protease-inactive S211A SepA (12) at 37°C for 30 min.

2D-DIGE analysis, including protein labeling, 2D electrophoreses, gel analysis, and identification of proteins of interest mass spectrometry analysis were performed by Applied Biomics (Hayward, CA) using established protocols. Specifically, Cy3 and Cy5 were used to label WT SepA-treated and S211A SepA-treated homogenized human colon preparations, respectively, and differences in migration were sought using 2-dimensional gel electrophoresis.

### AAT incubation with SepA, Pic, and SigA.

AAT (Sigma-Aldrich; A9024) was incubated with SepA, Pic, or SigA in PBS at 37°C for different periods of time, and the cleavage reactions were stopped by adding a 10-fold molar excess of PMSF. Reaction products were separated by sodium dodecyl-sulfate polyacrylamide gel electrophoresis (SDS-PAGE) on 4 to 20% precast polyacrylamide gels (Bio-Rad; 4561093) and silver stained (Bio-Rad; 1610449).

### Edman degradation.

Edman degradation analysis was performed at the Molecular Structure Facility (via Science Exchange) by following standard procedures.

### SepA, Pic, and SigA proteolytic activity assays.

As described previously (82), the synthetic substrate *N*-succinyl-Ala-Ala-Pro-Phe-*p*-nitroanilide (Suc-AAPF-pNA; S7388; Sigma-Aldrich) was used to evaluate the proteolytic activity of SepA. *N*-Succinyl-Ala-Ala-Pro-Abu-*p*-nitroanilide (Suc-AAP-Abu-pNA, SAP-3667-PI; Peptides International), a synthetic substrate previously shown to be efficiently cleaved by other SPATES (29), was used to analyze the proteolytic activities of Pic and SigA.

### Isolation of PMNs.

PMNs were isolated from whole blood obtained from normal healthy human volunteers by venipuncture as described previously (12, 83).

### Tissue culture.

T84 human intestinal epithelial cells were maintained in Dulbecco’s modified Eagle’s medium–F-12 supplemented with HEPES (15 mM; pH 7.5) and 10% fetal bovine serum. To obtain polarized monolayers, cells were grown in an inverted fashion on collagen-coated permeable polycarbonate transwells (Corning; 3388) and incubated at 37°C with 5% CO_2_, as described previously (12, 27, 28). Monolayers were used when cellular confluence and differentiation were achieved, usually after 7 to 8 days of inoculation and with the transepithelial electrical resistance of ≥1,200 Ω/cm^2^. All tissue culture reagents were obtained from Invitrogen.

### *In vitro* PMN migration assay.

A reaction mixture containing AAT (10 μM) and SepA (1 μM) was incubated in PBS at 37°C for 4 h and stopped by adding a 10-fold molar excess of PMSF. Logarithmic dilutions of this reaction mixture, corresponding to concentrations between 0.001 and 1,000 nM AAT initially added to the solution, were made and placed in plastic wells to evaluate their chemotactic activity. As described before, acellular collagen-coated transwells (84) or confluent T84 cell monolayers grown on transwells in an inverted fashion (12, 27, 28) were placed in wells containing different dilutions of the AAT-SepA reaction products. Freshly isolated human PMNs were then added to the upper compartment of each transwell. For acellular or transepithelial assays, samples were incubated at 37°C for 2 or 3 h, respectively. The number of migrated PMNs was determined via a colorimetric peroxidase assay, as previously described (12, 84). A 100 nM solution of fMLP and untreated medium (HBSS+) were also included as positive and negative controls of PMN migration, respectively.

### Combined PMN migration and *in vitro Shigella* invasion experiments.

Confluent T84 cell monolayers grown on transwells were apically infected with 5 × 10^6^ bacteria and incubated at 4°C for 30 min. Thereafter, the monolayers were transferred to wells with different concentrations of AAT in the lower (apical) compartment. Freshly isolated human PMNs were next added to the upper (basolateral) surface, and the transwells were incubated for 4.5 h at 37°C. PMNs remaining in the upper compartment of the transwells were recovered and counted via a colorimetric peroxidase assay. Transwells were further incubated in gentamicin (100 μg/ml) in both compartments for 3 h before the cells were washed with fresh HBSS+ and lysed with Triton X-100 (1%), as previously described (12). Gentamicin is an antibiotic that does not permeate eukaryotic plasma membranes and is therefore used to maintain only invading viable bacteria while removing extracellular populations of bacteria. To determine the number of invading bacteria, serial dilutions of each infected epithelial cell monolayer were performed and plated onto tryptic soy agar plates with 0.2% Congo red (27). Similarly, serial dilutions from the original bacterial inoculum used to infect the monolayers were also carried out. The number of CFU per condition was standardized by dividing the number of CFU obtained from the monolayers by the number of CFU in the inoculum. Results were normalized to values obtained from monolayers infected with WT S. flexneri (100%). Due to the high number of bacteria in the apical side, we performed an inverse measurement of PMN migration by subtracting the number of PMNs that remained in the upper compartment of the transwells from the initial amount added to the system. This method was validated by performing migration experiments with epithelial cell monolayers infected for 3 h at 37°C with WT S. flexneri or the avirulent, noninvasive BS103 strain. Following apical infection, epithelial cell monolayers were washed three times with fresh medium, PMNs were added to the basolateral surface, and they were incubated at 37°C for 3 more hours. Thereafter, the number of PMNs detected in both the apical and basolateral compartments were quantified, and the results were used to compare the traditional and inverse measurements of PMN migration (Fig. S5).

### Cytotoxicity assays.

The cytotoxic effects of AAT or the products of the AAT-SepA reaction were determined by the release of lactate dehydrogenase (LDH) from T84 cell monolayers or PMNs. T84 cell monolayers grown on 24-well plates or freshly isolated PMNs were incubated for 3 h in media containing different concentrations of AAT or the products of the AAT-SepA reaction. The supernatants were collected and used to evaluate the amounts of LDH released by using the LDH-Glo cytotoxicity assay (Promega; J2381) according to the manufacturer’s instructions.

### Statistical analysis.

Data are shown as means ± standard errors (SE). All statistical analyses were performed using GraphPad Prism 5 software. A 2-tailed nonparametric Mann-Whitney U test (MWUT) was used to calculate significant differences between treatment conditions. Asterisks are used to denote *P* values as follows: *, <0.05, and **, <0.01.

### Study approval.

Human colon collection was approved by the IRB of the University of Massachusetts Medical School Cancer Center Tissue Bank (IRB docket no. H1173). All neutrophil-based studies were approved by the IRB of the University of Massachusetts Medical School (protocol 13006). All subjects provided informed consent prior to participation in these studies.
