# Supplementary figures and images for: SepA Enhances Shigella Invasion of Epithelial Cells by Degrading Alpha-1 Antitrypsin and Producing a Neutrophil Chemoattractant
Source: mBio. 2021 Nov 2;12(6):e02833-21. doi: 10.1128/mBio.02833-21 (PMC8561385; doi:10.1128/mBio.02833-21)

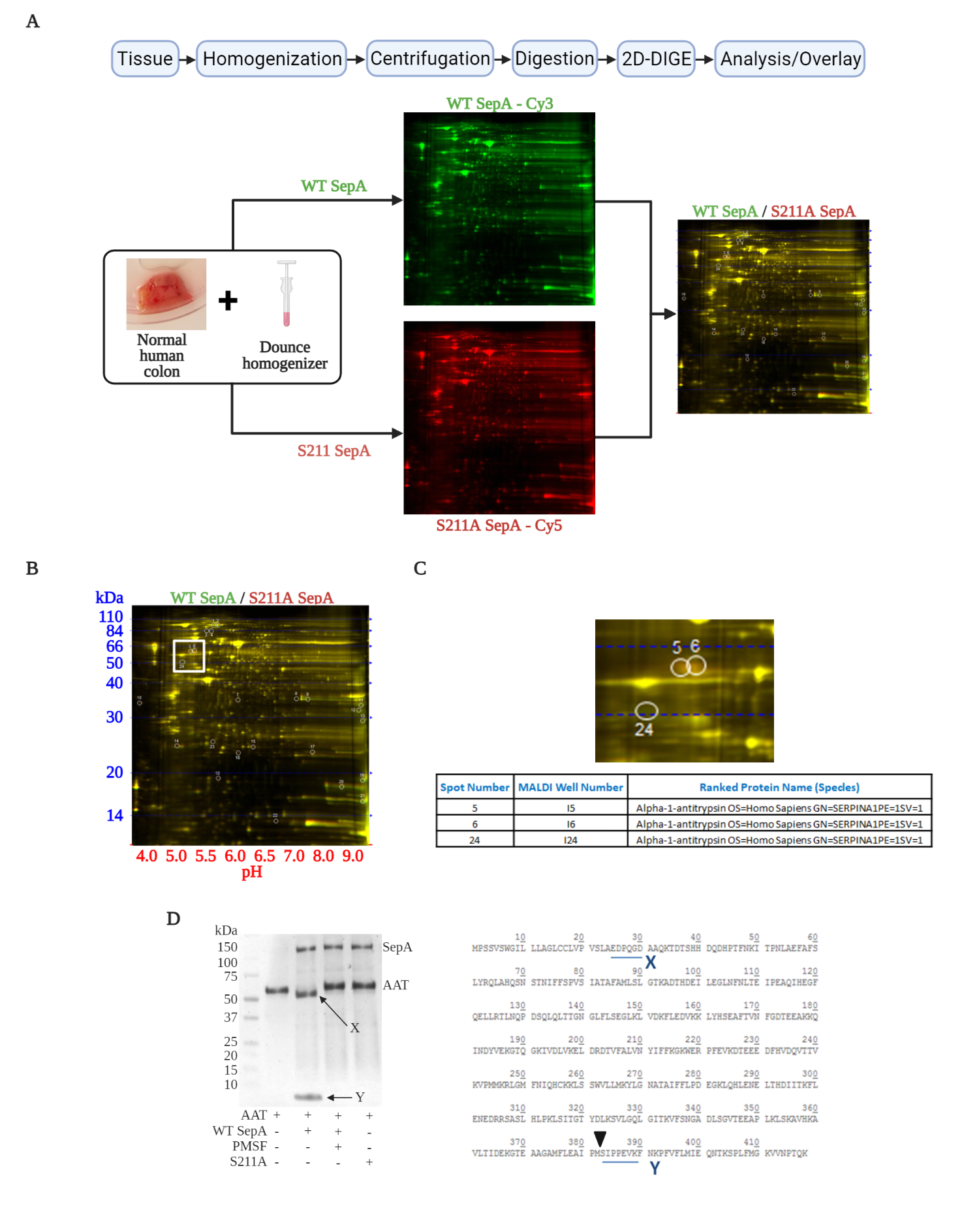

Supplement: FIG S1 [file mbio.02833-21-sf001.tif]

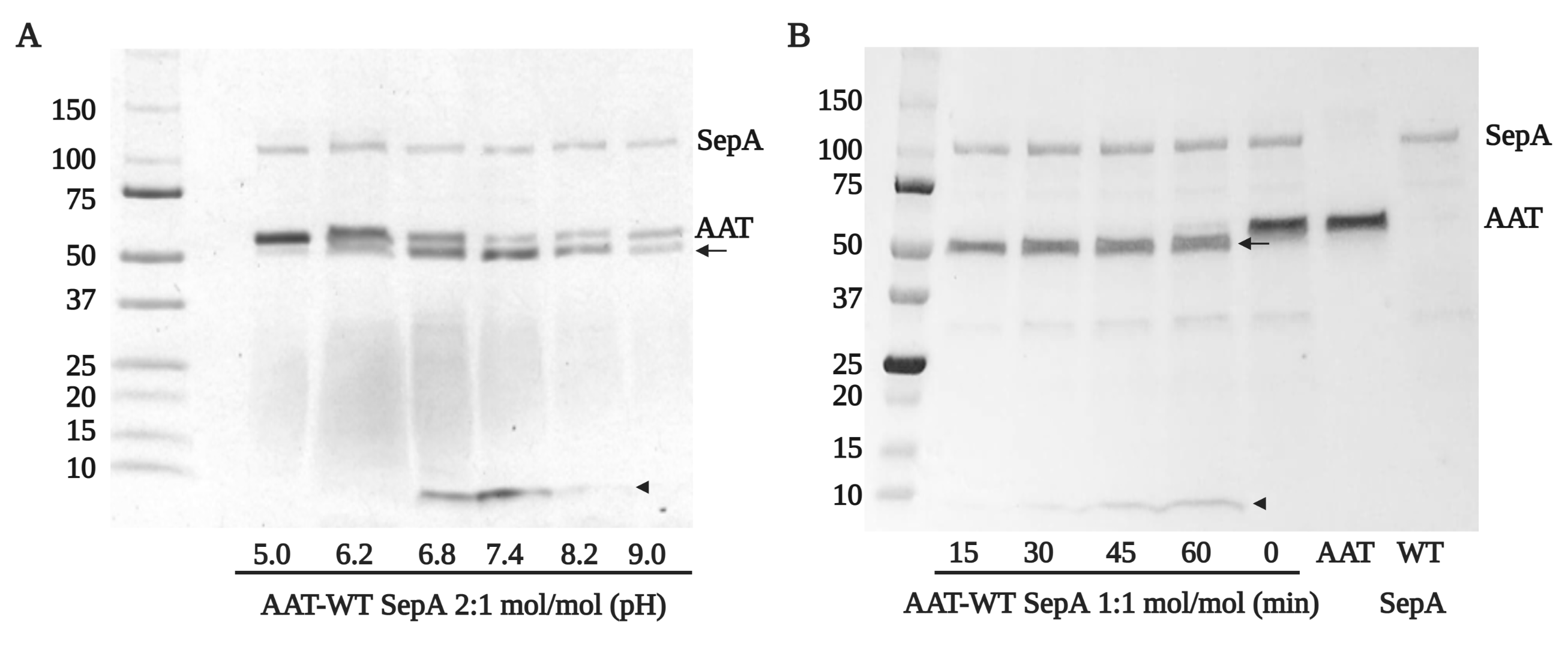

Supplement: FIG S2 [file mbio.02833-21-sf002.tif]

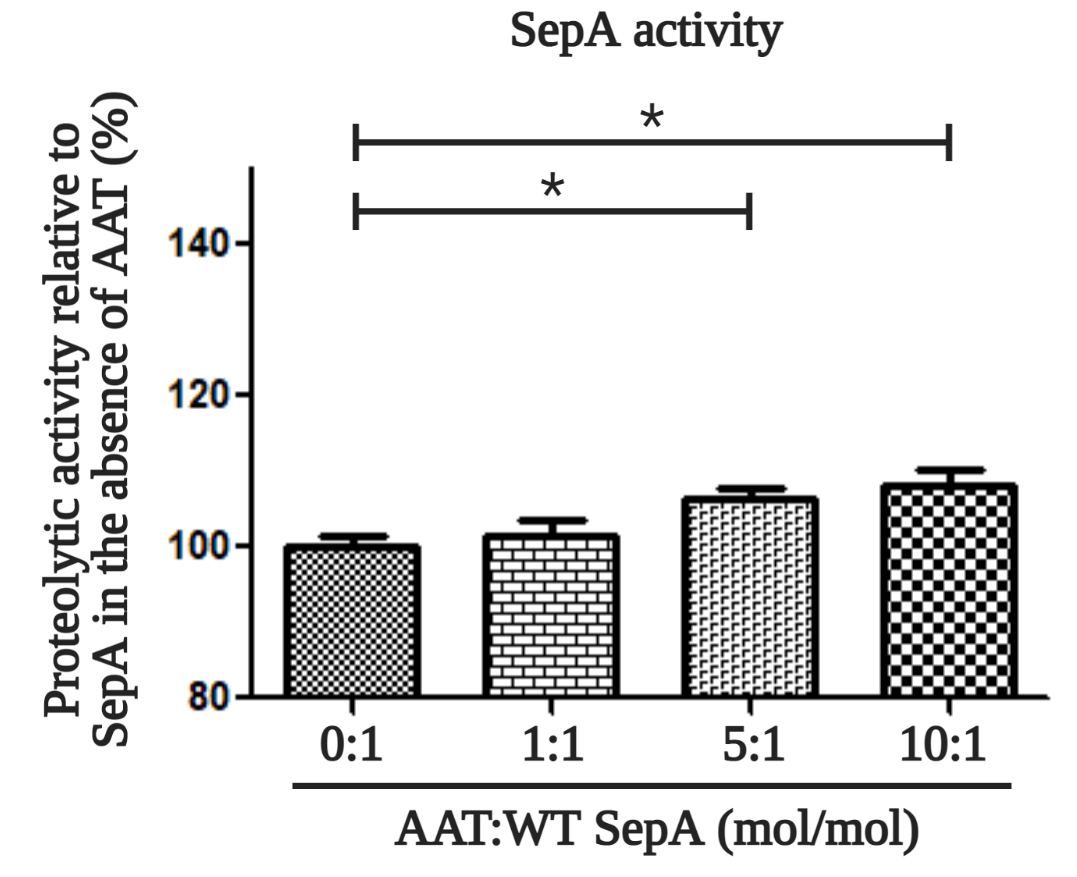

Supplement: FIG S3 [file mbio.02833-21-sf003.tif]

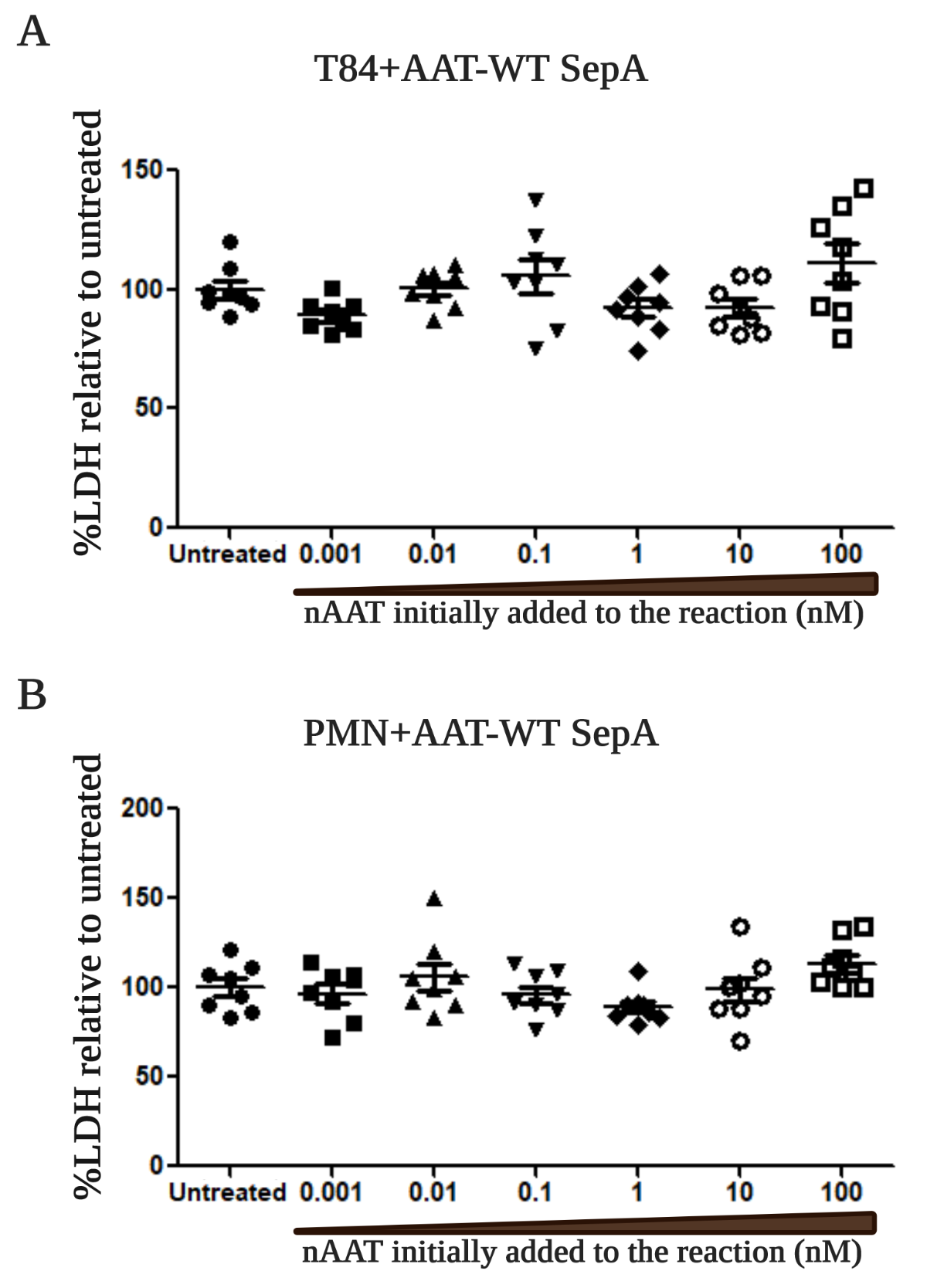

Supplement: FIG S4 [file mbio.02833-21-sf004.tif]

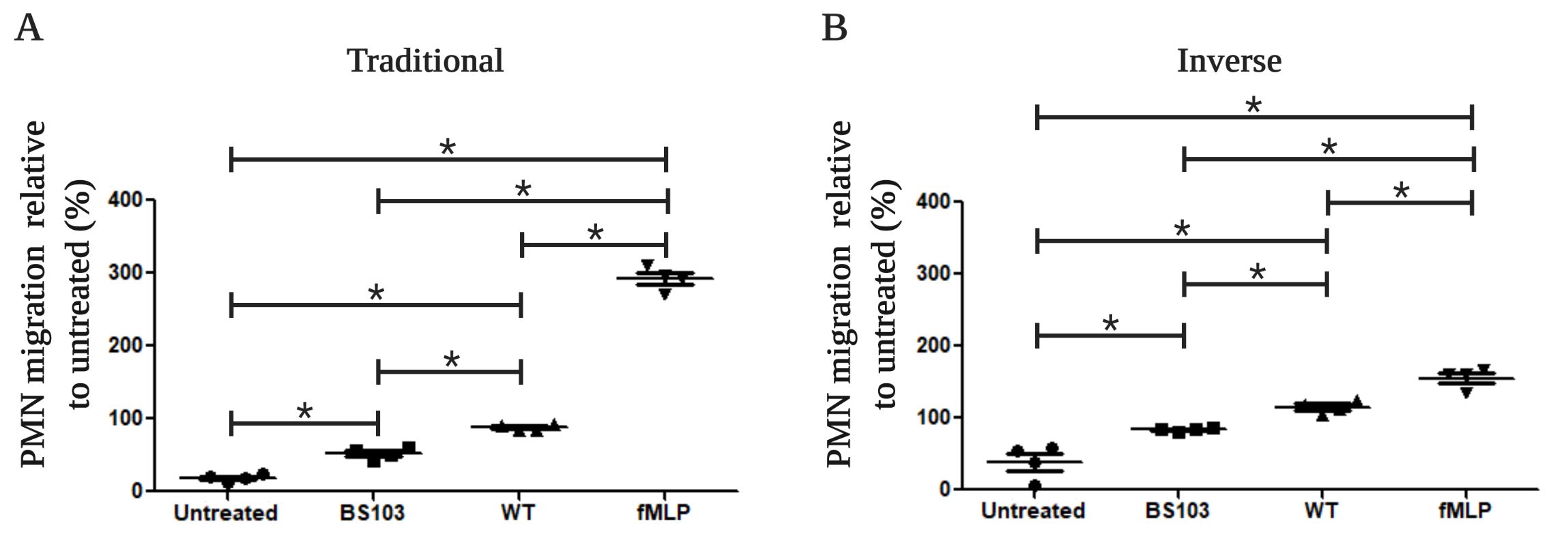

Supplement: FIG S5 [file mbio.02833-21-sf005.tif]

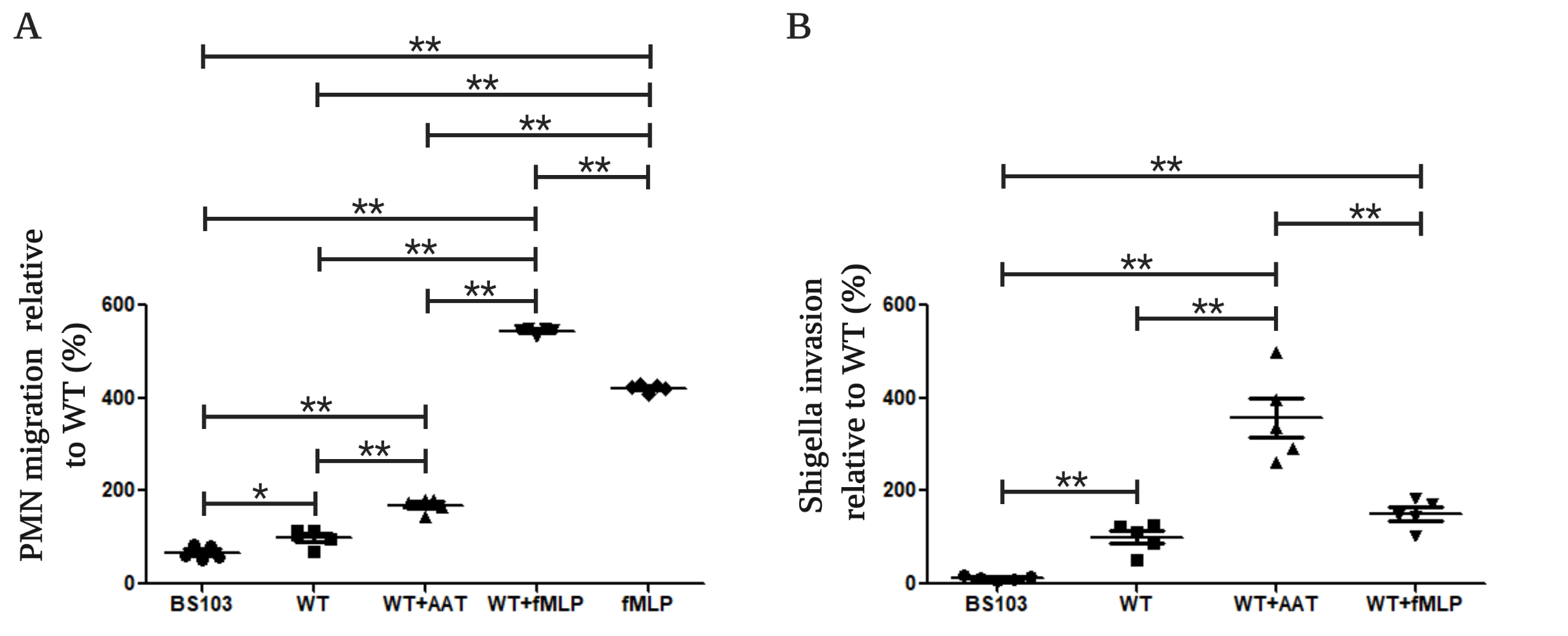

Supplement: FIG S6 [file mbio.02833-21-sf006.tif]

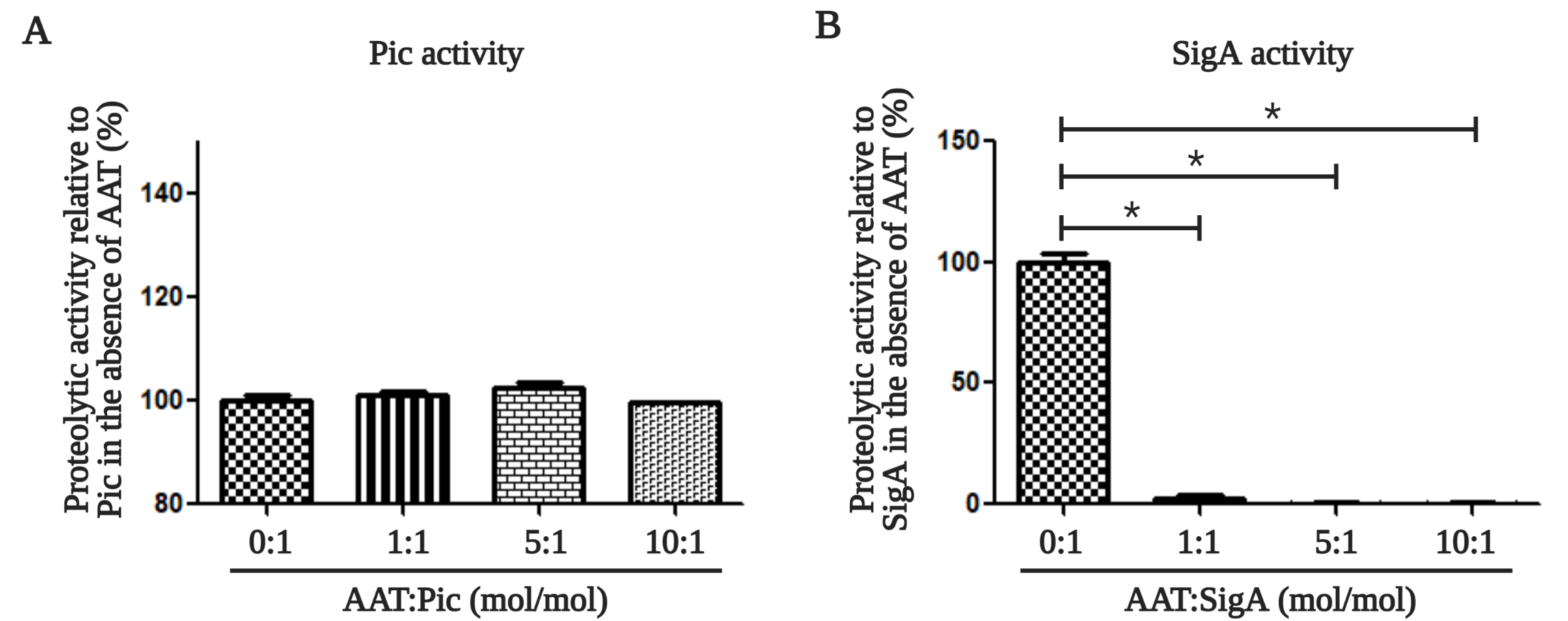

Supplement: FIG S7 [file mbio.02833-21-sf007.tif]
