# Supplementary material for: SepA Enhances Shigella Invasion of Epithelial Cells by Degrading Alpha-1 Antitrypsin and Producing a Neutrophil Chemoattractant
Source: mBio. 2021 Nov 2;12(6):e02833-21. doi: 10.1128/mBio.02833-21 (PMC8561385; doi:10.1128/mBio.02833-21)
Supplement: TABLE S1 [file mbio.02833-21-st001.docx]

**Table S1.** Strains and plasmids used in this work

| **Plasmids or strains** | **Description** | **Selection/Inducing agent** |
| --- | --- | --- |
| **Plasmids** |  |  |
| pZK15 | pUC19 containing *sep*A gene | Amp (100 µg/mL)/IPTG (0.5 mM) |
| pS211A | pUC19 containing *sep*A gene with a point mutation to change serine 211 to alanine | Amp (100 µg/mL)/IPTG (0.5 mM) |
| pPic1 | pUC19 containing *pic* gene | Amp (100 µg/mL)/IPTG (0.5 mM) |
| pSigA3 | pUC19 containing *sig*A gene | Amp (100 µg/mL)/IPTG (0.5 mM) |
| **Strains** |  |  |
| WT | Wild type *S. flexneri* 2a 2457TA | None |
| BS103 | WT-virulence plasmid cured | None |
| ∆*sep*A | WT with a *sep*A gene deletion | Cm (35 µg/mL) |
| ∆*sep*A+pZK15 | ΔsepA carrying pZK15 | Amp (100 µg/mL)/IPTG (0.5 mM) |
| BS103 ∆*pic*∆*sig*A | BS103 with *pic* and *sig*A deletions | Km (50 µg/mL) |
| BS103 ∆*pic*∆*sig*A pZK15 | BS103 ∆pic ∆sigA carrying pZK15 | Amp (100 µg/mL)/IPTG (0.5 mM) |
| BS103 ∆*pic*∆*sig*A S211A | BS103 ∆pic ∆sigA carrying S211A | Amp (100 µg/mL)/IPTG (0.5 mM) |
| BS103 ∆*pic*∆*sig*A pPic1 | BS103 ∆pic ∆sigA carrying pPic1 | Amp (100 µg/mL)/IPTG (0.5 mM) |
| BS103 ∆*pic*∆*sig*A pSigA3 | BS103 ∆pic ∆sigA carrying pSigA3 | Amp (100 µg/mL)/IPTG (0.5 mM) |
| ∆*sep*A∆*sig*A | WT with *sep*A and *sig*A gene deletions | Km (50 µg/mL) |
