# Supplementary material for: SepA Enhances Shigella Invasion of Epithelial Cells by Degrading Alpha-1 Antitrypsin and Producing a Neutrophil Chemoattractant
Source: mBio. 2021 Nov 2;12(6):e02833-21. doi: 10.1128/mBio.02833-21 (PMC8561385; doi:10.1128/mBio.02833-21)
Supplement: TABLE S2 [file mbio.02833-21-st002.docx]

**Table S2.** Primers used in this work

| **Description** | **Sense** | **DNA sequence(5' → 3')** |
| --- | --- | --- |
| Primers to delete *pic* | F | GGCATTTACACAGAATCTTAAACAGGCTCGCGCCAGTCGGCAAAACTAGTGTGTAGGCTGGAGCTGCTTC |
|  | R | CCTGGTGCCAATGATATTCCGGTATATGATAAGGACGGAAAACTTGTGGGATGGGAATTAGCCATGGTCC |
| Primers to delete *sig*A | F | TTCCCCGTTGTAATTAAATGCTATCCCATAACCACAACTCAGAAATATCGGTGTAGGCTGGAGCTGCTTC |
|  | R | ACAGGGTAATACATATTCTCTCCGGTAGAAGAAGGGCCGCAAACGCGGCCATGGGAATTAGCCATGGTCC |
| Primers to confirm *pic* deletion | F | AGTGATCTATACGACGAATGCC |
|  | R | GCTTCCCAACCTTACCAGAG |
| Primers to confirm *sig*A deletion | F | GTCGCACAGTTCCAGTTCAC |
|  | R | GCTTCCCAACCTTACCAGAG |
| Primers to clone *pic* | SphI-F | TATATCCCGCATGCACATCATGGAGAATCCATAGTG |
|  | XbaI-R | GAGTTCGGTCTAGACCTGGACGGTCACTTCTGAG |
| Primers to clone *sig*A | HindIII-F | CTGATCTGAAGCTTACCACAACTCAGAAATATCGG |
|  | XbaI-R | GAGTTCGGTCTAGACGGGCTGTTATCAGAAAGAG |
